# Synthesis of ruthenium oxide nanosheets with fourth-period elements for alkaline oxygen evolution reaction

**DOI:** 10.1080/14686996.2026.2722026

**Published:** 2026-08-24

**Authors:** Shuhei Dake, Saikat Bolar, Takeshi Fujita

**Affiliations:** School of Engineering Science, Kochi University of Technology, Tosayamada, Japan

**Keywords:** Oxygen evolution reaction, solid solution, ruthenium oxide, bimetallic oxide, rutile structure

## Abstract

A comprehensive series of Ru-based bimetallic oxide nanosheets with fourth-period elements (*M* = Cr, Mn, Fe, Co, Ni, Cu, Zn, and Ga) was synthesized via a unified puffing-template combustion method and systematically evaluated for the alkaline oxygen evolution reaction (OER). As revealed by X-ray diffraction, among the examined metals, Cr and Ga exclusively induced chemically selective solid-solution formation within the rutile RuO_2_ lattice, a structural prerequisite for modulating the electronic structure of the active Ru sites and enhancing the OER activity. The OER activity of the optimal compositions Ru_0.38_Ga_0.62_Ox and Ru_0.71_Cr_0.29_O_x_ surpassed that of pristine RuO_x_. However, a 50 h chronopotentiometric study revealed a decisive shift in the activity–stability relationship, with Ru_0.71_Cr_0.29_O_x_ maintaining a nearly constant operating potential, whereas Ru_0.38_Ga_0.62_O_x_ rapidly deactivated due to Ga^3+^ leaching. X-ray photoelectron spectroscopy and electron microscopy confirmed that Cr^3+^ acts as a valence-pinning agent, suppressing Ru oxidation, thereby reducing the density of oxygen vacancies and number of stable covalent Ru-O bonds. The results demonstrate that Ru_0.72_Cr_0.29_O_x_ is a promising and durable anode for the alkaline OER and highlight the importance of durability testing in concurrence with overpotential benchmarking.

## IMPACT STATEMENT

Selective Cr/Ga bimetallic oxide solid-solution formation in RuO_2_ from 4th period elements and introduces Cr^3+^-mediated valence pinning, a mechanism that simultaneously achieves benchmarking activity and high stability for oxygen evolution.

## GRAPHICAL ABSTRACT

**Figure:**
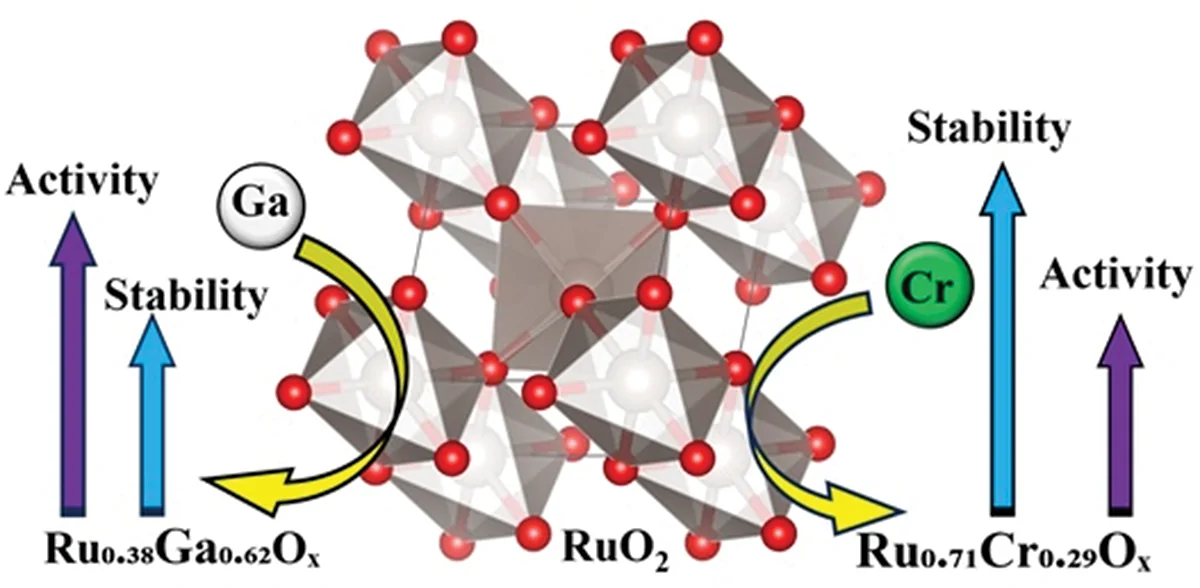

## 1. Introduction

Electrochemical water splitting has emerged as a cornerstone technique for green hydrogen production, offering a scalable pathway for decarbonizing the energy, industrial, and transport sectors [1]. The oxygen evolution reaction (OER), involving a thermodynamically demanding four-electron transfer process that imposes high intrinsic overpotential losses, remains the principal kinetic bottleneck [2]. The combination of sluggish OER kinetics and the absence of durable, cost-effective, high-activity catalysts continues to constrain the efficiency and commercial scalability of alkaline water electrolyzers (AWEs), in which the simultaneous requirements for high intrinsic activity, long term structural stability, and economic feasibility must be met [3,4].

Ruthenium dioxide (RuO_2_) remains the benchmark OER electrocatalyst, offering superior intrinsic activity at approximately 15% of the cost of iridium, together with strong oxygen-binding capacity and broad pH stability [5–7]. However, RuO_2_ suffers from a critical activity-stability trade-off under harsh anodic industrial conditions. Oxidative dissolution of lattice oxygen promotes over-oxidation of Ru^4+^ to soluble RuO_4_ species, thereby driving irreversible structural degradation [7,8]. Resolving this trade-off remains a central challenge in OER electrocatalysis. Two-dimensional (2D) ruthenate nanosheets have attracted considerable attention as electrocatalytic platforms owing to their high surface-to-volume ratios, enhanced active-site exposure, efficient mass transport ability, and the rapid diffusion of OH^−^ through their interlayer channels, thereby sustaining high current densities [9,10]. Their structural tunability renders them ideal candidates for compositional engineering via heteroatom doping and/or incorporation. Among these strategies, incorporating a secondary metal into the RuO_2_ rutile lattice has emerged as an effective approach for enhancing the OER activity while suppressing Ru dissolution [11]. Unlike surface decoration or physical mixing, solid-solution formation directly modulates the electronic structure of the Ru active sites through Ru-O-M bridging bonds. This modification alters the oxidation-state distribution of Ru, the covalent character of Ru–O bonds, and the adsorption free energies of key OER intermediates, including *OH, *O, and *OOH. Together, these effects can enhance the catalytic turnover frequency while improving resistance to Ru dissolution [12]. However, the performance requirements of OER catalysts vary considerably with the electrolyte environment. RuO_2_-based materials have been widely studied in acidic media, particularly for proton exchange membrane water electrolysis, where only a limited number of non-noble-metal oxides exhibit sufficient activity and stability. In alkaline media, by contrast, earth-abundant Ni-, Fe-, Co-, and NiFe-based catalysts often provide competitive activity at substantially lower cost. Accordingly, the present study does not seek to promote Ru-based oxides as low-cost catalysts for alkaline OER. Instead, it employs a unified Ru–M oxide platform to systematically clarify how the incorporation of fourth-period metals influences rutile solid-solution formation, electronic structure, alkaline OER activity, and long-term durability.

Fourth-period transition and post-transition metals provide a particularly diverse compositional space for designing bimetallic oxides. Their systematic variations in d-orbital occupancy, ionic radius, electronegativity, and oxophilicity can strongly influence the local structure and electronic environment of Ru sites when incorporated into an oxide lattice [13]. Although heteroatom incorporation into rutile RuO_2_ has been extensively explored using elements spanning nearly 45% of the periodic table, a unified comparison of the complete fourth-period metal series within a consistently synthesized Ru–M oxide platform under identical alkaline OER conditions has not yet been reported [14,15]. In the absence of such a systematic evaluation, the selection of secondary metals for alkaline water electrolysis remains largely dependent on fragmented results obtained using different synthesis methods and testing protocols, making reliable cross-material comparisons difficult.

Among fourth-period candidates, Cr^3+^ has attracted mechanistic attention as a Lewis acid site that enhances OH^−^ adsorption and accelerates the generation of *O radicals in Cr_x_Ru_1-x_O_2_ solid solutions, while contraction of the Ru-Cr bond stabilizes the Ru-O framework against anodic dissolution [11]. Gallium, with a stable oxidation state (+3 state) and strong oxophilicity, acts as a fundamentally distinct electronic modifier, where oxophilic Ga^3+^ sites incorporated into the Ru-O lattice are expected to accelerate hydroxyl adsorption and water dissociation in alkaline media [11,16]. Nevertheless, a controlled, comparative study of the catalytic activity and long-term stability of Ru-Ga versus Ru-Cr bimetallic oxides under equivalent conditions has not been reported.

Most studies exclusively employ polarization curves and brief chronoamperometric holds as durability proxies, an approach insufficient for industrial AWE, where anodes must sustain continuous high-current-density operation over thousands of hours [17,18]. A catalyst that achieves low overpotential may, nonetheless, undergo progressive degradation via metal leaching, phase segregation, or crystallographic amorphization, thereby limiting its suitability for practical use. To resolve this distinction, the activity and stability of catalysts must be evaluated on a unified basis, a standard not consistently applied to Ru-M bimetallic oxide systems [19].

To address these gaps, we report the synthesis and systematic evaluation of the structural performance of a comprehensive Ru-M bimetallic oxide series that spans the entire 4th period (*M* = Cr, Mn, Fe, Co, Ni, Cu, Zn, and Ga), all prepared via a single, unified synthetic protocol to ensure that observed differences are attributable solely to the identity of the secondary metal. The structure of the catalysts is characterized and correlated with the performance in the alkaline OER in 1 M KOH. The long-term stability of the catalysts is assessed by chronopotentiometry at industrially relevant current densities. Among the investigated catalysts, Ru-Cr oxide and Ru-Ga oxide exhibited the highest alkaline OER activity, with both outperforming benchmark RuO_2_. Ru-Ga showed the lower overpotential and Tafel slope during short-term electrochemical measurements, indicating faster initial reaction kinetics. However, prolonged chronopotentiometric testing revealed that Ru-Cr retained substantially better structural integrity and electrochemical performance. This superior durability identifies Ru-Cr as the more promising anode material for practical alkaline water electrolysis. The contrasting activity and stability trends also highlight the importance of evaluating electrocatalysts using multiple performance indicators rather than relying solely on initial overpotential or kinetic parameters.

To the best of our knowledge, this study provides the first systematic investigation of the structure-activity relationships of Ru-based bimetallic oxides incorporating fourth-period elements for alkaline OER. The complete catalyst series was synthesized using a consistent method and evaluated in 1 M KOH to establish clear relationships among composition, crystal structure, catalytic activity, and long-term stability under conditions relevant to alkaline water electrolysis. However, acidic OER measurements were not included because the present catalyst library was specifically designed for alkaline operation and was not optimized for stability in strongly acidic environments. A reliable assessment under acidic conditions would require separate catalyst optimization, extended durability measurements, and quantitative dissolution analysis. Therefore, the findings of this study apply specifically to alkaline OER conditions.

## 2. Experimental

### 2.1. Materials

Glucose (C_6_H_12_O_6_), ammonium nitrate (NH_4_NO_3_), chromium(III) chloride hexahydrate (CrCl_3_·6 H_2_O), manganese(II) chloride tetrahydrate (MnCl_2_·4 H_2_O), iron(II) chloride tetrahydrate (FeC_2_·4 H_2_O), cobalt(II) chloride hexahydrate (CoCl_2_·6 H_2_O), nickel(II) chloride hexahydrate (NiCl_2_·6 H_2_O), copper(II) chloride dihydrate (CuCl_2_·2 H_2_O), zinc chloride (ZnCl_2_), and gallium nitrate hydrate (Ga(NO_3_)_3_·xH_2_O) were used as received from Wako Pure Chemical Industries, Osaka, Japan. Ruthenium(III) chloride hydrate (RuCl_3_·xH_2_O) was sourced from Sigma-Aldrich, U.S.A. All solutions were prepared using ultrapure deionized water (Millipore Milli-Q, U.S.A.).

### 2.2. Synthesis of Ru-M oxide nanosheets

**Figure 1.**
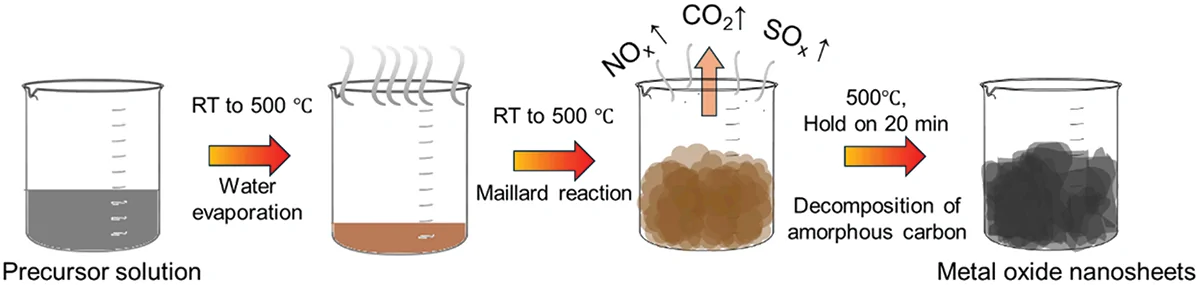
Schematic of combustion synthesis of puffing-template of Ru-M oxide nanosheets (*M* = Cr, Mn, Fe, Co, Ni, Cu, Zn, and Ga).

Glucose (2.0 g), NH_4_NO_3_ (2.5 g), and Ru + M metal salt precursors (100 mg total metal, nominal 1:1 Ru:M atomic ratio) were dissolved in deionized water (20 mL) in a quartz beaker and ultrasonicated for 1 min to produce a homogeneous precursor solution. The beaker was placed in an electric muffle furnace, heated to 500°C at a controlled rate of approximately 32°C min^−1^, and held at 500°C for 20 min to drive the combustion synthesis of the puffing template. The furnace was then switched off, and the product was allowed to cool naturally at 25°C, affording Ru-M oxide nanosheets (Figure 1). The glucose/NH_4_NO_3_ system functions simultaneously as a carbon template, fuel, and oxidizer. Rapid gas evolution during combustion generates the nanosheet morphology, while the exothermic redox reaction between the organic fuel and the nitrate oxidizer drives the in-situ formation of metal oxides at applied temperature.

### 2.3. Physicochemical characterization

The crystal structure and phase composition of the samples were investigated by powder X-ray diffraction (XRD; Rigaku SmartLab SE, Cu Kα radiation, Japan). The surface morphology and microstructure were examined by field-emission scanning electron microscopy (FE-SEM; Hitachi SU8600, Japan) and transmission electron microscopy (TEM; JEOL NEOARM, Japan). The elemental composition and spatial distribution were determined using energy-dispersive X-ray spectrometry (EDS) coupled with FE-SEM and TEM. High-resolution transmission electron microscopy (HRTEM), fast Fourier-transform (FFT) analysis, and selected-area electron diffraction (SAED) were used to assess the crystallographic integrity at the atomic scale. The surface chemical states and electronic structure were evaluated via X-ray photoelectron spectroscopy (XPS) measurements carried out at the Institute for Materials Research, Tohoku University, using an Al Kα radiation source and X-ray monochromator (AXIS ultra DLD, Shimadzu, Japan). The specific surface area of Ru_0_._38_Ga_0_._62_O_x_ and Ru_0_._71_Cr_0_._29_O_x_ were determined by N_2_ physisorption using a BELSORP-max II gas sorption analyzer (MicrotracBEL Corp., Japan).

### 2.4. Electrochemical measurements

All electrochemical measurements were performed in 1 M KOH using an Ivium Vertex workstation (Ivium Technologies B.V., The Netherlands) in a conventional three-electrode configuration with a catalyst-coated glassy carbon rotating disc electrode (GC-RDE, 5 mm diameter, geometric area: 0.196 cm^2^) as the working electrode, Pt wire as the counter electrode, and Ag/AgCl (saturated KCl) as the reference electrode. All potentials were converted to the scale of the reversible hydrogen electrode (RHE) using the following equation [20]:

(1) $$E_{\mathrm{RHE}}=E_{\mathrm{Ag}/A\mathrm{gCl}}+0.197+0.0591\times \mathrm{pH}$$

The data were *iR*-corrected using the uncompensated resistance (*R_u_*), determined via electrochemical impedance spectroscopy (EIS).

(2) $$E_{\mathrm{corrected}}=E-iR_{u}$$

Catalyst inks were prepared by dispersing 5 mg of Ru-M oxide powder in a mixed solvent of deionized water (250 μL), ethanol (750 μL), and 5 wt% Nafion solution (25 μL), followed by probe ultrasonication for 30 min. For the linear sweep voltammetry (LSV) analyses, 10 μL of ink was drop-cast onto a GC-RDE and dried at room temperature (catalyst loading: 0.25 mg cm^−2^); LSV data were recorded at 5 mV s^−1^ for the reference samples and at 10 mV s^−1^ for the Ru-M bimetallic series to minimize interference owing to the accumulation of O_2_ bubbles. For the durability assessments, catalyst ink was deposited onto carbon paper substrates (0.2 cm × 1.0 cm; loading: 0.50 mg cm^−2^), and the long-term stability was evaluated by chronopotentiometry at a constant current density of 10 mA cm^−2^ over 50 h in 1 M KOH.

## 3. Results and discussion

**Figure 2.**
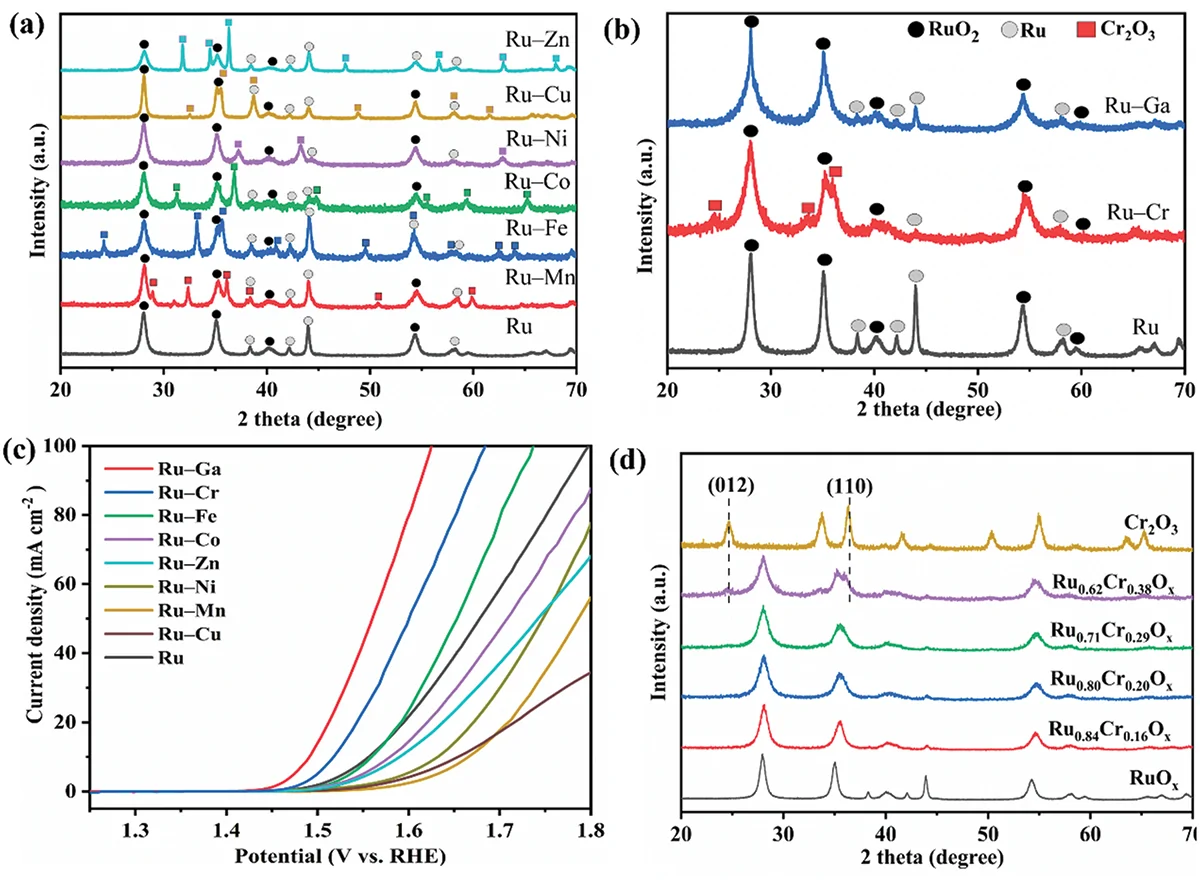
(a) XRD patterns of Ru-M oxide nanosheets (*M* = Mn, Fe, Co, Ni, Cu, Zn) and Ru reference; peaks indexed to rutile RuO_2_ (●), metallic Ru (○), and secondary M-oxide phases (■), indicating phase segregation. (b) XRD patterns of RuCr and Ru-Ga confirming single-phase rutile solid-solution for Ru-Ga and predominantly rutile structure with minor Cr_2_O_3_ traces (■) for Ru-Cr. (c) LSV polarization curves in 1 M KOH, demonstrating superior OER activity of Ru-Ga and Ru-Cr over all other bimetallic and Ru reference samples. (d) Composition-dependent XRD patterns of the Ru-Cr system; emergence of Cr_2_O_3_ (012) and (110) reflections establish a solid-solution solubility limit of ~29 at% Cr.

The bimetallic Ru-M oxide nanosheets (*M* = Mn, Fe, Co, Ni, Cu, and Zn), along with a Ru reference, were characterized by powder XRD (Figure 2(a)). The reflections of the RuO_2_ reference are assignable to hexagonally close-packed metallic ruthenium, indicating that direct combustion of the Ru precursor in the absence of a secondary metal yields the metallic phase. Upon introduction of fourth-period transition metals, the XRD patterns of all six bimetallic samples show reflections characteristic of the rutile-type RuO_2_ structure (●), demonstrating that the secondary metal promotes Ru oxidation during combustion. However, peaks assignable to secondary metal oxide phases (■) MnO_x_ Fe_2_O_3_, CoO_x_, NiO, CuO, and ZnO, respectively, are also detected for all samples, without measurable shifts of the rutile peaks. The co-existence of distinct RuO_2_ and M-oxide reflections with no change in the lattice parameters is diagnostic of phase segregation, in which the secondary metals fail to substitute into the rutile lattice and instead crystallize as discrete oxide phases [20–22]. This outcome is consistent with the large mismatches in the ionic radii, electronegativity, and preferred coordination geometry among late first-row transition-metal cations and the Ru^4+^ rutile host, which collectively prevent thermodynamically stable solid-solution formation under the current synthesis conditions [21,22].

Figure 2(b) presents a comparison of the XRD patterns of Ru-Cr and Ru-Ga with that of the Ru reference. The diffractogram of the Ru-Ga sample shows rutile-type RuO_2_ reflections with no detectable Ga_2_O_3_ or Ga(OH)_3_ peaks, confirming complete incorporation of Ga^3+^ into the rutile lattice to form a single-phase solid-solution oxide. The pattern of the Ru-Cr sample exhibits dominant RuO_2_ reflections, accompanied by low-intensity Cr_2_O_3_ peaks (■), indicating that Cr is incorporated largely but not completely in the nominal 1:1 Ru:Cr ratio [23]. These results demonstrate that among the eight fourth-period metals examined, only Ga and Cr form solid solutions with rutile RuO_2_ under puffing-template combustion conditions (Table S1). The preferential incorporation of Cr^3+^ and Ga^3+^ is rationalized by their +3 oxidation state (compatible with Ru^4+^ charge compensation via oxygen vacancy formation or mixed valency), similar octahedral ionic radii (*r*_Cr_^3+^ = 0.615 Å; *r*_Ga_^3+^ = 0.620 Å vs. *r*_Ru_^4+^ = 0.620 Å), and pronounced oxophilicity, which favors metal-oxygen bonding within the rutile framework [11,16,23,24].

To delineate the limit of Cr solubility within the rutile lattice, XRD was used to evaluate the composition-dependent changes (Figure 2(d)). At Cr loadings up to Ru_0.71_Cr_0.29_O_x,_ only rutile reflections appear in the diffractograms, confirming the formation of a single-phase solid solution. At Ru_0.62_Cr_0.38_O_x_, the characteristic (012) and (110) reflections of Cr_2_O_3_ clearly emerge, confirming supersaturation-driven phase separation [22,23]. Thus, the Cr solubility limit is approximately 29 at%, as corroborated by EDS compositional analysis (Figure S1). Collectively, the structural data in Figure 2(a–d) demonstrate that the formation of a solid solution with rutile RuO_2_ is chemically selective, restricted to Ga and Cr among the fourth-period metals and that the Ru-Cr system accommodates up to ~29 at% Cr before phase segregation occurs. Furthermore, conventional XRD analysis cannot entirely exclude the presence of small amounts of amorphous or poorly crystallized components in the Ru-M oxide nanosheets. Therefore, the XRD patterns are interpreted as evidence of the dominant crystalline phases rather than as confirmation that all disordered species are absent. The lack of additional diffraction peaks suggests that no XRD detectable segregated crystalline oxide phases are present, although trace amorphous surface species below the detection limit may still exist.

The alkaline OER activity of all synthesized materials was evaluated by LSV in 1 M KOH (Figure 2(c)). Among the bimetallic systems, a clear activity hierarchy emerges across the fourth period, with Ru-Ga showing the lowest onset potential and the highest current response, followed by Ru-Cr as the second most active catalyst. The remaining bimetallic samples (Ru-Fe, Ru-Co, Ru-Zn, Ru-Ni, Ru-Mn, and Ru-Cu) display progressively diminished OER activity in approximately this order, with Ru-Cu performing the worst among the bimetallic series, as also confirmed by the Tafel slopes (Figure S2).

The superior performance of Ru-Ga and Ru-Cr is directly correlated with their unique ability to form solid-solution rutile structures, as established by the XRD data (Figure 2(a,b)). In phase-segregated systems, the secondary metal oxide exists as a discrete phase that does not electronically perturb Ru^4+^ within the rutile lattice. The OER activity is, therefore, governed predominantly by isolated RuO_2_ domains, with only minor contributions from M-oxide interfaces. In contrast, direct substitution of Cr^3+^ or Ga^3+^ into the rutile framework induces systematic electronic perturbation of the adjacent Ru sites through Ru-O-M bridging bonds, modulating the distribution of Ru oxidation states, covalency of the Ru-O bonds, and adsorption free energies of the key OER intermediates (*OH, *O, *OOH), thereby lowering the effective energy barrier for the rate-determining step [12,23,25]. The activity ranking (Ru-Fe > Ru-Co > Ru-Zn ≈ Ru-Ni > Ru-Mn > Ru-Cu) broadly reflects the intrinsic alkaline OER activities of the corresponding metal oxides, consistent with independent phase contributions rather than synergistic electronic coupling.

The enhanced short-term activity of Ru-Ga compared to that of Ru-Cr can be rationalized by the pronounced oxophilicity and d^0^ electronic configuration of Ga^3+^. Ga^3+^ lacks partially filled d-orbitals and thus acts as a purely structural and electrostatic modifier of the Ru-O lattice, with no competing d-electron interactions [25]. Ga^3+^ substitution for Ru^4+^ in the rutile framework induces charge-compensating enhancement of the electron density at the adjacent Ru sites through Ru-O-Ga back-donation, lowering the free energy for *OH adsorption and facilitating the first proton-coupled electron-transfer step in the alkaline OER mechanism [11,16,25]. Additionally, the pronounced oxophilicity of Ga^3+^ accelerates hydroxyl adsorption and water dissociation at the Ga site, cooperatively accelerating the OER kinetics [11,24]. Cr^3+^, functioning as a Lewis acid center within the rutile lattice, enhances OH^−^ adsorption and promotes the generation of *O radicals, while the shortened Ru-Cr interatomic distance stabilizes the Ru-O framework [11,24]. Tafel slope analysis (Figure S2) further confirms that in the short-term, Ru-Ga exhibits superior kinetics compared to Ru-Cr.

Having established that Ru-Ga and Ru-Cr are the only fourth-period systems capable of solid rutile solution formation (Figure 2(a,b)), composition-dependent analyses were performed to identify the Ga and Cr loadings at which the alkaline OER activity was maximal within each series.

**Figure 3.**
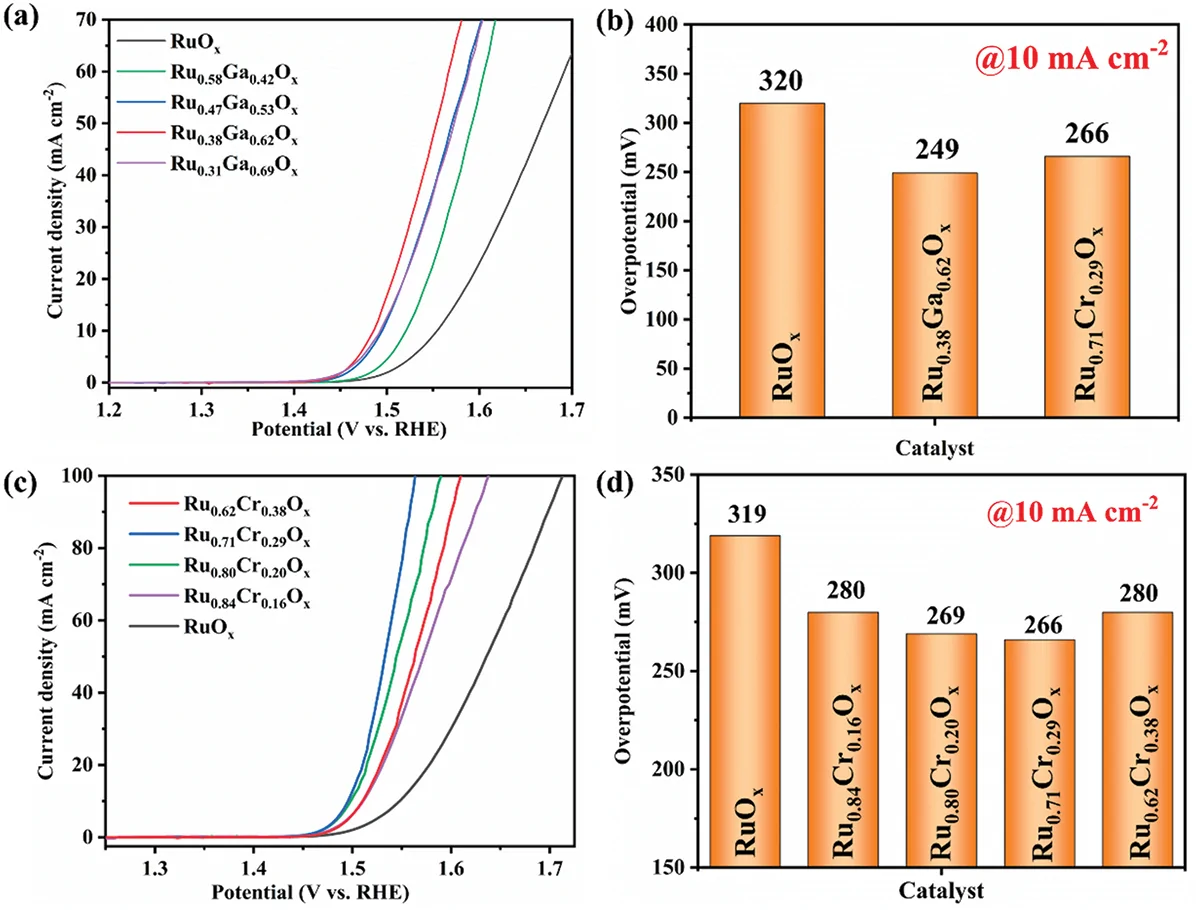
(a) Composition-dependent LSV polarization curves of the Ru-Ga series in 1 M KOH at 10 mV s^−1^. (b) Overpotentials at 10 mA cm^−2^ for the Ru-Ga series. (c) Composition-dependent LSV curves for Ru-Cr series in 1 M KOH at 10 mV s^−1^. (d) Overpotentials at 10 mA cm^−2^ for Ru-Cr series.

The LSV polarization curves and corresponding overpotentials at 10 mA cm^−2^ (η_10_) are shown in Figure 3(a–d), respectively. Compared to the RuO_x_ reference (η_10_ = 325 mV), progressive Ga^3+^ incorporation monotonically reduces the overpotential to 291, 266, and 256 mV for Ru_0.58_Ga_0.42_O_x_, Ru_0.47_Ga_0.53_O_x_, and Ru_0.38_Ga_0.62_O_x_, respectively. The overpotential reaches a minimum for Ru_0.38_Ga_0.62_O_x_, representing a 69 mV improvement compared to that of undoped RuO_x_ and the lowest overpotential recorded across the entire fourth-period series. Further addition of Ga to Ru_0.31_Ga_0.69_O_x_ causes a slight activity reversal (η_10_ = 264 mV). The monotonic improvement up to Ru_0.38_Ga_0.62_O_x_ reflects progressive electronic promotion with increasing Ga^3+^ substitution, which enhances the electron density at adjacent Ru sites via Ru-O-Ga back-donation, lowering the free energy for *OH adsorption [11,16,20,26]. The activity reversal at Ru_0.31_Ga_0.69_O_x_ arises from two competing effects: (i) progressive dilution of the electrochemically active Ru sites below the threshold required to sustain cooperative O-O radical coupling, which reduces the geometric turnover frequency; and (ii) lattice distortion beyond the elastic limit of the solid-solution framework, which introduces structural disorder that impedes electron transport within the electrode film [23]. The Ru_0.38_Ga_0.62_O_x_ composition mitigates these effects by providing the optimal balance between Ga-mediated electronic promotion and the density of Ru active sites.

A structurally informative activity profile can be deduced from the series of Ru-Cr compositions (Figure 3(c,d)). Interestingly, with increasing Cr content, the overpotential (η_10_) gradually decreases from 280 mV for Ru_0.84_Cr_0.16_O_x_ to 269 mV for Ru_0.80_Cr_0.20_O_x_, reaching a minimum of 266 mV for Ru_0.71_Cr_0.29_O_x_, representing a 53 mV improvement over RuO_x_. Beyond this composition, η_10_ rises sharply to 280 mV for Ru_0.62_Cr_0.38_O_x_ owing to the onset of phase segregation between the RuO_2_ and Cr_2_O_3_ domains, which disrupts the homogeneous Ru-Cr-O solid-solution structure and eliminates the synergistic electronic coupling between the Ru and Cr centers, which is responsible for the optimized d-band occupancy and enhanced OER activity at lower Cr loadings [23,24]. Critically, the optimal electrochemical activity (Ru_0.71_Cr_0.29_O_x_) coincides exactly with the XRD-determined Cr solubility limit in the rutile lattice (~29 at%), whereas the activity-deteriorating composition (Ru_0.62_Cr_0.38_O_x_) exceeds this limit, and clear Cr_2_O_3_ reflections are observed in the XRD pattern (Figure 2(d), Figure S3). This direct correspondence constitutes compelling mechanistic evidence that the formation of a single-phase solid solution is a structural prerequisite for maximizing the OER activity of the Ru-Cr system [11]. Once the solubility limit is exceeded and Cr_2_O_3_ precipitates as a segregated phase, the Ru-O-Cr electronic coupling is disrupted. The resulting Cr_2_O_3_ phase, being a wide-bandgap insulator with negligible intrinsic OER activity in alkaline media, physically blocks the Ru active sites and hinders charge transfer, which directly increases the required potential [24]. Tafel and EIS analysis further corroborate the superior kinetics of the reaction employing Ru_0.71_Cr_0.29_O_x_ over Ru_0.38_Ga_0.62_O_x_ and undoped RuO_x_ (Figure S4 & S5). The improved kinetic behavior is evidenced by a lower Tafel slope and reduced charge-transfer resistance, indicating faster interfacial electron transfer and more efficient OER intermediate conversion. This improvement is likely associated with Ru-O-Cr electronic coupling, which optimizes the local electronic environment of Ru active sites and promotes alkaline OER kinetics.

A direct comparison of the two optimized compositions, Ru_0.38_Ga_0.62_O_x_ (η_10_ = 256 mV) and Ru_0.71_Cr_0.29_O_x_ (η_10_ = 266 mV), shows that Ru-Ga exhibits a modest 10 mV activity advantage under short-term polarization conditions (Figure S6 and Table 2). The Ru-Ga system tolerates a substantially higher optimal metal loading (62 at% Ga vs. 29 at% Cr at the Ru-Cr solubility limit), reflecting the higher compatibility of d^0^ Ga^3+^ with the rutile lattice, because Ga^3+^ lacks crystal-field stabilization energy (CFSE) that would otherwise impose substitution constraints, compared to d^3^ Cr^3+^, which is subject to octahedral CFSE [25]. Although Ru_0.38_Ga_0.62_O_x_ emerges as the most active catalyst in short-term polarization benchmarking, comprehensive evaluation for industrial AWE must extend to long-term operational stability.

**Figure 4.**
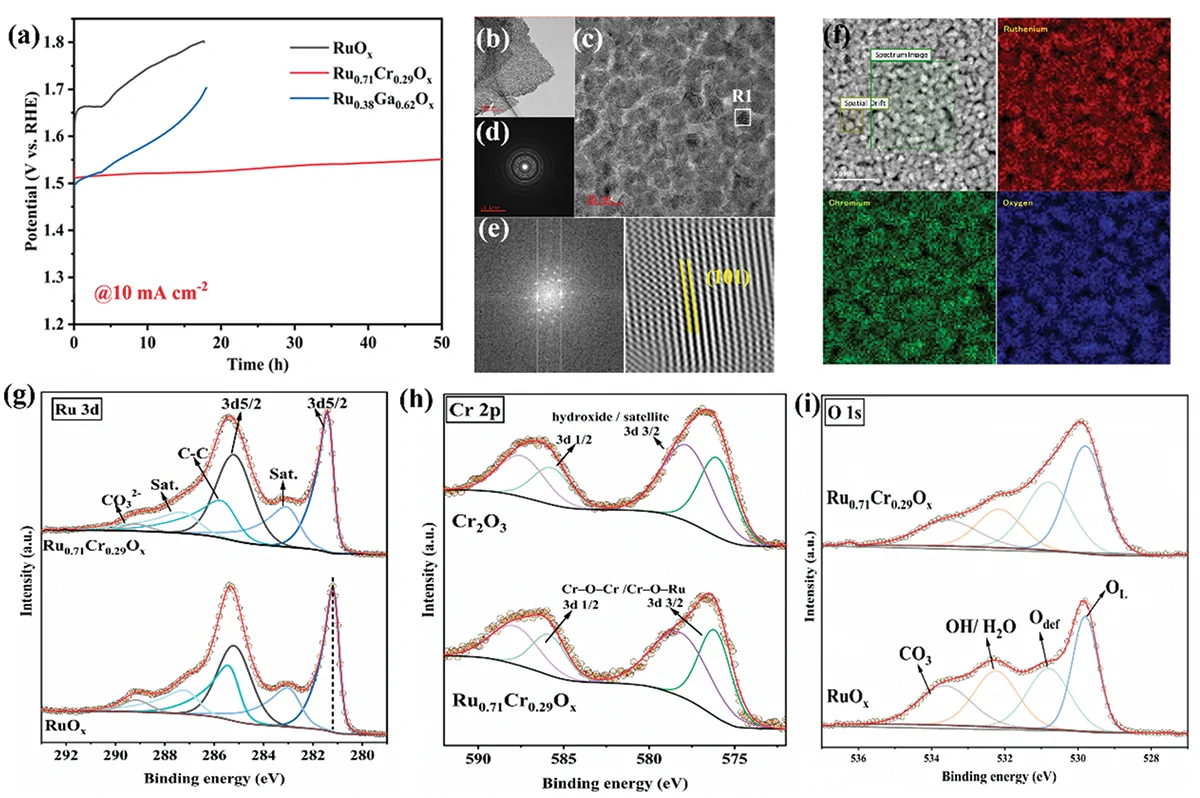
(a) Chronopotentiometric stability profiles of RuO_x_, Ru_0_._71_Cr_0_._29_O_x_, and Ru_0_._3_8Ga_0_._62_O_x_ at 10 mA cm-2 over 50 h. (b-e) TEM, HRTEM/FFT, and SAED analyses of Ru_0_._71_Cr_0_._29_O_x_ confirming phase-pure rutile solid-solution structure. (f) STEM image and EDS elemental maps demonstrating uniform distribution. (g-i) XPS profiles of Ru 3d, Cr 2p, and O1s core levels.

Long-term chronopotentiometric stability tests at 10 mA cm^−2^ over 50 h in 1 M KOH (Figure 4(a)) present a fundamentally different picture from the short-term polarization benchmarking. The RuO_x_ reference undergoes the most severe degradation, where its operating potential rises sharply from ~1.65 V vs. RHE at onset to ~1.80 V within 20 h, characteristic of progressive dissolution of the active phase and irreversible structural collapse, driven by the formation of volatile RuO_4_ species under sustained anodic conditions [7,8,27,28]. Despite its superior short-term activity, the potential of Ru_0.38_Ga_0.62_O_x_ increases significantly and continuously throughout the chronopotentiometric test, rising from ~1.50 V to ~1.70 V vs. RHE within 35 h, with a degradation rate far exceeding that of Ru_0.71_Cr_0.29_O_x_. In stark contrast, Ru_0.71_Cr_0.29_O_x_ maintains a remarkably stable operating potential of ~1.50–1.52 V vs. RHE throughout the 50 h window, with negligible potential drift, the hallmark of structural and electrochemical integrity under sustained anodic operation. The contrasting trends in short-term activity (Ru–Ga > Ru–Cr) and long-term stability (Ru–Cr > Ru–Ga > RuO_x_) highlight the well-known activity–stability trade-off that remains a central challenge in Ru-based OER electrocatalysis [17–19,19]. Despite its slightly lower initial activity, Ru_0.71_Cr_0.29_O_x_ exhibited the best comparative stability when evaluated under identical alkaline OER conditions using carbon-paper-supported electrodes.

The mechanistic origin of this divergence is rooted in the fundamentally different crystallographic–chemical roles of Cr^3+^ and Ga^3+^ as modifiers of the rutile lattice. Cr^3+^ (d^3^, t_2_g^3^) occupies the octahedral sites of rutile, with a substantial crystal-field stabilization energy (CFSE = −1.2Δo for the d^3^ configuration in an octahedral ligand field), providing a thermodynamic driving force for Cr to remain within the rutile coordination environment under prolonged anodic polarization and resist leaching into the electrolyte [25]. The resulting Ru-O-Cr bridging framework imparts structural rigidity to the rutile lattice, suppressing the oxygen-vacancy-mediated dissolution pathway by which Ru^4+^ is oxidized to soluble RuO_4_ [11,27–29]. In contrast, Ga^3+^ (d^0^) derives zero CFSE from the octahedral rutile site. Under prolonged exposure to the alkaline conditions at the anode, Ga^3+^ is susceptible to dissolution as the thermodynamically stable gallate anion Ga(OH)_4_^−^ and to structural rearrangement that progressively disrupts Ru-O-Ga coupling [11,16,28]. The continuous increase in the potential observed for Ru_0.38_Ga_0.62_O_x_ is attributed to gradual Ga^3+^ leaching, concurrent loss of electronic promotion at the Ru sites, re-exposure of the less-active bare Ru sites, and progressive amorphization of the nanosheet framework.

The HRTEM image of Ru_0.71_Cr_0.29_O_x_ (Figure 4(b–e)) displays well-resolved periodic lattice fringes with a d-spacing consistent with the (101) planes of rutile RuO_2_, confirming that the Ru-Cr solid-solution retains the rutile crystal structure on the atomic scale. The corresponding FFT spot pattern (Figure 4(e)) can be indexed exclusively to the rutile phase, with no reflections attributable to Cr_2_O_3_ or any secondary phase. The intensity profile recorded along the [101] direction displays regular periodic oscillations with consistent spacing and amplitude, confirming well-defined crystallographic ordering within the analyzed region. The SAED pattern (Figure 4(d) and Figure S7) exhibits distinct concentric diffraction rings that can be indexed to the rutile RuO_2_ structure, with no additional reflections attributable to metallic Ru, Cr_2_O_3_, or other crystalline impurity phases. Furthermore, STEM–EDS elemental mapping (Figure 4(f)) shows a uniform spatial distribution of Ru, Cr, and O throughout the nanosheet, without noticeable compositional gradients or Cr-rich segregated regions. These observations strongly support the formation of a homogeneous Ru–Cr solid-solution structure [23]. Collectively, the TEM, HRTEM, SAED, and EDS results confirm that Ru_0.71_Cr_0.29_O_x_ forms a highly crystalline and compositionally homogeneous rutile solid solution without detectable phase segregation. This well-ordered structure, together with the nanosheet morphology, is likely to contribute to the superior chronopotentiometric stability observed in Figure 4(a) by improving resistance to anodic dissolution compared with structurally disordered or phase-separated counterparts [8].

To further verify the nanosheet architecture and assess the accessible surface area of the optimized catalysts, N_2_ adsorption-desorption measurements were performed. Ru_0.38_Ga_0.62_O_x_ and Ru_0.71_Cr_0.29_O_x_ exhibited BET specific surface areas of 123.70 and 78.00 m^2^ g^−1^, respectively. These relatively high surface areas are consistent with the formation of sheet-like oxide structures produced by rapid gas evolution during the puffing-template combustion process. Together with the TEM observations, the textural results provide complementary evidence for the nanosheet-like morphology of the Ru-M oxide catalysts (Figure S8).

High-resolution XPS data were acquired for the core-level Ru 3d, Cr 2p, and O 1s regions to elucidate the surface-chemical basis of the superior stability of Ru_0.71_Cr_0.29_O_x_ (Figure 4(g–i)). The Ru 3d spectrum shows a positive shift of the Ru XPS peaks after Cr incorporation, indicating electron depletion around the Ru sites owing to strong electronic interaction between the Ru and Cr species (Figure 4(g)). Namely, the incorporation of Cr modulates the Ru 4d electronic structure through Ru-O-Cr coupling, which raises the binding energy of the Ru species. The contributions from higher-valence Ru species (Ru^4+δ^, Ru^6+^) and shake-up satellites are proportionally suppressed relative to those in RuO_x_, demonstrating that Cr^3+^ incorporation stabilizes Ru in the +4 oxidation state and suppresses over-oxidation. XPS analysis reveals that suppression of higher Ru valence states plays a critical role in enhancing the chronopotentiometric stability of Ru_0.71_Cr_0.29_O_x_. Ru dissolution follows the sequential oxidation pathway Ru^4+^ → Ru^6+^ → RuO^4^, and Cr^3+^ functions as a valence-pinning species that limits excessive Ru oxidation under anodic polarization; these features suppress dissolution and improve the long-term stability of the material [27–29].

Deconvolution of the Cr 2p spectrum of Ru_0.71_Cr_0.29_O_x_ reveals components that can be assigned to Cr-O-Cr and Cr-O-Ru bridging environments. The presence of a distinct Cr-O-Ru component, absent in the pure Cr_2_O_3_ reference, provides unambiguous spectroscopic evidence that Cr^3+^ is covalently integrated into the Ru-O lattice framework rather than existing as a segregated Cr_2_O_3_ phase [23,29]. The binding energy of Cr 2p_3/2_ in Ru_0.71_Cr_0.29_O_x_ is shifted to slightly lower values relative to that in Cr_2_O_3_, consistent with the increased electron density on Cr arising from back-donation by Ru-O-Cr (Figure 4(h)). The exclusive oxidation state of Cr^3+^ and the complete absence of Cr^6+^ (~579.5 eV, such as CrO_4_^2-^ or CrO_3_) corroborate the claim that Cr does not undergo anodic oxidation, confirming its role as a chemically inert structural and electronic stabilizer rather than as a redox-active OER participant [11,29].

The O 1s spectra provide complementary information about the chemistry of oxygen on the sample surface (Figure 4(i)). The O 1s spectrum of RuO_x_ shows peaks of four resolved components: lattice O^2-^ (~529.7 eV), hydroxyl/defect oxygen bridge (~531.0 eV), surface OH (~532.3 eV), and adsorbed molecular water (~533.8 eV). The large contributions from surface OH and adsorbed water confirm a defect-rich, heavily hydroxylated surface with abundant under-coordinated Ru sites prone to oxidative dissolution. In Ru_0.71_Cr_0.29_O_x_, the lattice O^2-^ component becomes the overwhelmingly dominant species, while the contributions of surface OH and adsorbed water are markedly suppressed. This redistribution has two critical mechanistic implications: (i) the enhanced oxygen fraction in the lattice confirms that Cr^3+^ incorporation reduces the density of oxygen vacancies and promotes the formation of a crystallographically ordered M-O-M lattice in which the metal is more fully coordinated, directly limiting the vacancy-mediated dissolution nucleation mechanism [8,19,29]; and (ii) suppression of excess surface hydroxyl groups indicates a cleaner active surface, with OH species present at levels that promote the formation of OER intermediates rather than forming a deactivating passive layer [26].

The superior durability of Ru_0.71_Cr_0.29_O_x_, therefore, arises from at least four synergistic stabilization mechanisms operating simultaneously: (i) CFSE-driven thermodynamic anchoring of Cr^3+^ within the octahedral site of rutile, which prevents Cr leaching and maintains Ru-O-Cr electronic coupling throughout prolonged operation [11,25]; (ii) Cr^3+^ valence-pinning of Ru in the +4 oxidation state, which directly interrupts the Ru^4+^ → Ru^6+^ → RuO_4_ dissolution cascade confirmed by Ru 3d XPS (Figure 4(g)) [7,8,27–29]; (iii) reduction of the oxygen-vacancy density and lattice-defect concentration, which suppresses the vacancy-mediated dissolution nucleation mechanism established by O 1s XPS (Figure 4(i)) [19,29]; and (iv) covalent Ru-O-Cr bridging bonds, as confirmed by Cr 2_p_ XPS (Figure 4(h)), which ensures uniform stabilization of the Ru sites across the electrode surface and prevents preferential dissolution adjacent to unmodified domains [23,29].

Post-test characterization was conducted to clarify the possible degradation pathways following chronopotentiometric testing. The XRD patterns of the tested catalysts showed no significant changes compared with those of the pristine samples, indicating that the dominant rutile solid-solution structure was largely preserved after alkaline OER operation (Figure S9). This structural retention demonstrates the stability of the Ru-M oxide framework under the applied electrochemical conditions. In particular, Ru_0.71_Cr_0.29_O_x_ retained its nanosheet-like morphology and rutile solid-solution structure, while Ru and Cr remained uniformly distributed throughout the catalyst, as confirmed by post-test microscopy and EDS analyses (Figures S10-S12). In contrast, Ru_0.38_Ga_0.62_O_x_ showed more pronounced structural degradation accompanied by a weaker Ga signal, suggesting that partial Ga leaching may have occurred during alkaline OER (Figures S10–S13). The RuO_x_ control showed the most pronounced degradation, consistent with the progressive oxidation and dissolution of Ru species under prolonged anodic polarization. These results indicate that the enhanced stability of Ru_0.71_Cr_0.29_O_x_ arises primarily from Cr-induced stabilization of the rutile lattice rather than from any stabilizing contribution of the carbon-paper substrate.

In addition, UV-vis analysis of the post-test electrolyte showed no characteristic absorption attributable to soluble Cr species within the detection limit of the measurement (Figure S14). Together with the post-test XRD, microscopy, and EDS results, this observation further supports the compositional and structural stability of the Ru-Cr solid-solution catalyst during alkaline OER.

Although Ru_0.71_Cr_0.29_O_x_ exhibited superior comparative stability during 50 h of chronopotentiometric testing in 1 M KOH, practical alkaline water electrolysis involves considerably harsher operating conditions, including concentrated KOH, elevated temperatures, high current densities, and prolonged anodic polarization. Therefore, the present stability test should be considered a comparative screening assessment rather than a complete demonstration of industrial durability. Further evaluation under practically relevant conditions, such as 30 wt% KOH at 80°C in a membrane-electrode assembly or alkaline electrolyzer configuration, will be necessary to verify catalyst robustness, electrode-level stability, and suitability for long-term operation.

## 4. Conclusions

This study presents the first comprehensive evaluation of the structure-performance relationship for a complete fourth-period Ru-M bimetallic oxide series, where *M* = Cr, Mn, Fe, Co, Ni, Cu, Zn, and Ga, for the alkaline OER using a unified puffing template combustion synthesis protocol. This systematic approach enables direct comparison of the catalysts without synthetic bias and reveals that phase structure selectivity is a key factor governing alkaline OER activity. Among the eight secondary metals examined, only Cr and Ga were incorporated into the rutile RuO_2_ lattice to form solid solution oxides under the present synthesis conditions. In contrast, Mn, Fe, Co, Ni, Cu, and Zn formed phase segregated composites, in which the secondary metal oxides crystallized as discrete phases and provided limited synergistic electronic modulation of Ru active sites. These results identify solid-solution formation as a structural prerequisite for enhancing alkaline OER activity in this Ru-M oxide system. Both Ru_0.38_Ga_0.62_O_x_ and Ru_0.71_Cr_0.29_O_x_ exhibited higher alkaline OER activity than pristine RuO_x_, achieving η_10_ values of 256 and 266 mV, respectively. The superior short-term activity of Ru_0.38_Ga_0.62_O_x_ is attributed to the d^0^ electronic configuration and strong oxophilicity of Ga^3+^, which electronically modifies the Ru-O framework and facilitates hydroxyl adsorption. In the Ru-Cr system, the optimum activity coincides with the Cr solubility limit of approximately 29 at%, demonstrating that a phase pure Ru-O-Cr solid solution framework is essential for maximizing the catalytic advantage. Once this solubility limit is exceeded, Cr_2_O_3_ segregation occurs, blocking Ru active sites and impeding charge transfer. Long-term chronopotentiometric testing revealed a decisive reversal in the activity-stability relationship. Although Ru_0.38_Ga_0.62_O_x_ showed the lowest initial overpotential, it underwent continuous deactivation during prolonged alkaline OER. In contrast, Ru_0.71_Cr_0.29_O_x_ maintained a nearly stable operating potential with negligible drift, indicating markedly superior durability. This enhanced stability is attributed to Cr^3+^ incorporation into the rutile lattice, which stabilizes the Ru-O framework, suppresses excessive Ru oxidation, reduces oxygen vacancy related degradation, and maintains covalent Ru-O-Cr bonding during anodic operation. The instability of Ru_0.38_Ga_0.62_O_x_ is associated with the susceptibility of d^0^ state of Ga^3+^ to progressive leaching under alkaline anodic conditions, which weakens the Ru-O-Ga electronic promotion effect and accelerates catalyst degradation.

Overall, this systematic fourth-period investigation establishes Ru_0.71_Cr_0.29_O_x_ as a promising anode catalyst for alkaline OER, combining competitive activity with superior long-term stability. More broadly, the contrasting behavior of Ru-Ga and Ru-Cr demonstrates that short term activity alone is not sufficient to assess the practical viability of Ru-based OER catalysts. A rigorous catalyst evaluation must therefore integrate activity benchmarking with long-term durability analysis to identify materials with genuine potential for alkaline water electrolysis applications.

## Data Availability

The data will be made available on reasonable request.
